# Biological and immunological characterization of recombinant Yellow Fever 17D Viruses expressing a *Trypanosoma cruzi *Amastigote Surface Protein-2 CD8^+ ^T cell epitope at two distinct regions of the genome

**DOI:** 10.1186/1743-422X-8-127

**Published:** 2011-03-18

**Authors:** Raquel T Nogueira, Alanderson R Nogueira, Mirian CS Pereira, Maurício M Rodrigues, Ricardo Galler, Myrna C Bonaldo

**Affiliations:** 1 Fundação Oswaldo Cruz, Instituto Oswaldo Cruz, Laboratório de Biologia Molecular de Flavivírus, Rio de Janeiro, Fundação Oswaldo Cruz. Avenida Brasil 4365, Manguinhos, Rio de Janeiro, RJ, 21045-900, Brazil; 2 Fundação Oswaldo Cruz, Instituto de Tecnologia em Imunobiológicos, Rio de Janeiro, Brazil; 3 Fundação Oswaldo Cruz, Instituto Oswaldo Cruz, Laboratório de Ultra-estrutura Celular, Rio de Janeiro, Brazil; 4 Centro de Terapia Celular e Molecular (CTCMol), Universidade Federal de São Paulo-Escola Paulista de Medicina, Rua Mirassol, 207, São Paulo-SP 04044-010, Brazil

## Abstract

**Background:**

The attenuated Yellow fever (YF) 17D vaccine virus is one of the safest and most effective viral vaccines administered to humans, in which it elicits a polyvalent immune response. Herein, we used the YF 17D backbone to express a *Trypanosoma cruzi *CD8^+ ^T cell epitope from the Amastigote Surface Protein 2 (ASP-2) to provide further evidence for the potential of this virus to express foreign epitopes. The TEWETGQI CD8^+ ^T cell epitope was cloned and expressed based on two different genomic insertion sites: in the *fg *loop of the viral Envelope protein and the protease cleavage site between the NS2B and NS3. We investigated whether the site of expression had any influence on immunogenicity of this model epitope.

**Results:**

Recombinant viruses replicated similarly to vaccine virus YF 17D in cell culture and remained genetically stable after several serial passages in Vero cells. Immunogenicity studies revealed that both recombinant viruses elicited neutralizing antibodies to the YF virus as well as generated an antigen-specific gamma interferon mediated T-cell response in immunized mice. The recombinant viruses displayed a more attenuated phenotype than the YF 17DD vaccine counterpart in mice. Vaccination of a mouse lineage highly susceptible to infection by *T. cruzi *with a homologous prime-boost regimen of recombinant YF viruses elicited TEWETGQI specific CD8^+ ^T cells which might be correlated with a delay in mouse mortality after a challenge with a lethal dose of *T. cruzi*.

**Conclusions:**

We conclude that the YF 17D platform is useful to express *T. cruzi *(Protozoan) antigens at different functional regions of its genome with minimal reduction of vector fitness. In addition, the model *T. cruzi *epitope expressed at different regions of the YF 17D genome elicited a similar T cell-based immune response, suggesting that both expression sites are useful. However, the epitope as such is not protective and it remains to be seen whether expression of larger domains of ASP-2, which include the TEWETGQI epitope, will elicit better T-CD8+ responses to the latter. It is likely that additional antigens and recombinant virus formulations will be necessary to generate a protective response.

## Background

The Yellow Fever Virus (YF) is a member of the *Flavivirus *genus and *Flaviviridae *family. The YF genome consists of a single positive-stranded RNA molecule with an approximate 11 kb length encoding a single polyprotein precursor. The YF polyprotein is processed by cellular and viral proteases generating the viral structural proteins which compose the virus particle, namely capsid (C), membrane (M) and its precursor (prM) plus envelope (E) in addition to the non-structural proteins NS1, NS2A, NS2B, NS3, NS4A, NS4B and NS5, possessing different roles in viral replication [1].

The attenuated yellow fever (YF) 17D vaccine is one of the safest and most effective attenuated viral vaccines available for human immunization. Its production, under strict quality control procedures, has been administered to man since the late 1930's [2]. A single prime dose promotes an excellent seroconversion rate in more than 90% of all vaccinees and can provide immunity for more than 30 years, yielding a robust and persistent neutralizing antibody response as a primary adaptive defense [3]. A role for cell-mediated immunity driven by a single YF 17D virus vaccine dose was first proposed [4] and in addition confirmed with the identification of YF-specific human effector and memory T CD8^+ ^cells addressed to E, NS1, NS2B and NS3 proteins of YF 17D [5-7]. However, understanding of the mechanisms by which the YF 17D virus triggers immune response is only now being unveiled and includes a multiple of virus component interactions with the immune system. The YF 17D virus was shown to induce a polyvalent immune response due to its capacity to infect and activate different subsets of human dendritic cells, via Toll-like receptors (TLRs), resulting in the production of pro-inflammatory cytokines, such as interferon α (IFN-α) and other interleukins (IL-12p40, IL-6), thus the basis to generate the marked adaptive immune response succeeding YF 17D virus vaccination [8]. Adaptive immune response to YF 17D virus immunization is characterized by a considerable expansion of specific activated T CD8^+ ^cells together with a mixed T helper cell (Th1 and Th2) cytokine profile controlled by stimulation of different TLRs [9,10]. These results indicate a relevant immunological starting point for the characterization of recombinant YF 17D viruses as new vaccine candidates, suggesting they resemble YF 17D in its natural immune response.

For more than ten years, YF 17D has been developed as a recombinant viral vector to express other flavivirus proteins, such as the prM/E of Japanese Encephalitis Virus, Dengue Virus and West Nile Virus [11]. While these 17D recombinants are based on the substitution of equivalent YF 17D genes, other antigens from unrelated pathogens have also recently been successfully expressed and delivered by the recombinant YF 17D as alternative strategies for genetic manipulation of the YF 17D genome. One of the previous studies described a recombinant YF 17D virus containing an ovalbumin immunodominant cytotoxic T cell epitope, expressed at the junction of NS2B and NS3 genes, which was cleaved of the polyprotein due to the duplication of viral protease NS2B/3 cleavage sites [12]. The same YF cloning strategy was employed in a subsequent work to express a *Plasmodium yoelli *CD8^+ ^T cell epitope [13]. Both of these studies demonstrated that YF 17D recombinant viruses do protect vaccinated mice against lethal challenge with malignant melanoma cells or the malaria parasite, respectively. Another possibility of YF 17D engineering was developed by our group through three-dimensional structure modeling analysis of the YF E protein with the identification of two expression sites permissive to heterologous insertions [14,15]. A number of recombinant YF 17D viruses that express T cell and humoral epitope of the *Plasmodium spp *CS protein inserted at the *fg *loop have been constructed. These viruses were quite stable through serial passages in cultured vertebrate cells and also retained attenuation for mice and monkeys [16]. The epitope expression in the E protein seems to be an interesting approach, since there have been recent reports that this protein elicits dominant immune responses corresponding either to MHC class I or class II antigens [17,18].

In this study, we have evaluated the expression of an immunodominant epitope of ASP-2 (amastigote surface protein 2) of *Trypanosoma cruzi*, the ethiological agent of Chagas disease, by the YF 17D virus. *T.cruzi *is an obligate intracellular protozoan parasite. Despite recent efforts to reduce transmission in Latin America, Chagas disease still affects near 13 million people, provoking more than 15,000 deaths per year. Another 50 to 80 million people are at risk of infection [19]. Recently, the occurrence of microepidemics due to food borne transmission has been reported [20]. In this regard, new strategies should be adopted in order to develop an efficacious prophylaxis for Chagas disease.

ASP-2 is a 83 kDa surface protein of *T. cruzi *intracellular amastigote forms, recognized by specific cytotoxic T lymphocytes of mice and man [21,22]. It has been described as one of the best antigen targets for a Chagas vaccine [23-25]. Previous reports on immunization of highly susceptible As/n mice with an ASP-2 recombinant plasmid, protein or viral vectors have exhibited protection against a lethal *T. cruzi *infection in conjunction with Th1 adjuvants or heterologous prime-boost regimens. However, the depletion of CD8+ T cells in vaccinated animals prior the challenge with *T.cruzi *caused high mortality rates. It was demonstrated that the protective CD8 + T lymphocytes were directed to the H-2K^k^-restricted immunodominant epitope TEWETGQI positioned from 320 to 327 amino acid residues of ASP-2 [25]. Although the immunization with TEWETGQI elicited similar numbers of peptide-specific IFN-γ secreting cells as did recombinant ASP-2 protein fragments containing the immunodominant octapeptide, protective immunity after T.cruzi challenge could be only elicited by the immunization with the TEWETGQI epitope in the context of a ASP-2 protein fragments [26]. The lack of protective immunity observed following immunization TEWETGQI was correlated with a lower priming of memory peptide-specific CD8+ T cells [26].

YF-17D virus is one the most efficient human vaccine virus ever obtained. It is also an outstanding inducer of CD8+ T cell immune responses [6]. We aimed at defining whether in the context of the YF 17D vector the immunodominant ASP-2 epitope, TEWETGQI, could elicit CD8^+ ^T cell response and protective immunity against a lethal challenge with *T. cruzi*. To determine that the TEWETGQI epitope was expressed at two different locations of the YF 17D genome, that is, between NS2B and NS3 proteins and at the E protein dimer surface.

We also intended to compare the efficacy of these two strategies in eliciting specific T cell responses against this *T.cruzi *epitope, since the cell compartments involved in the expression and release of the foreign epitope are distinct. The TEWETGQI epitope is expected to be released in the cell cytoplasm when expressed at the NS2B/NS3 site and in the ER as part of the envelope protein, which will eventually form the viral envelope used to assemble virus particles and exiting the cell through its secretory pathway. In addition, we characterized these recombinant viruses in terms of replication and attenuation. Both recombinant viruses were also evaluated in relation of their ability to induce protection against a lethal challenge with *T. cruzi *.

## Results

### Generation of YF 17D recombinant viruses expressing a *T. cruzi *T-cell epitope

**Figure 1:**
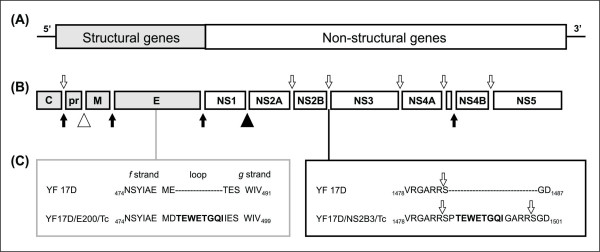
**Schematic diagram of YF 17D virus genome organization and the corresponding insertion sites**. The YF virus genome (A) is translated in a polyprotein of 3,411 amino acids (B), which is proteolytically processed by the viral protease complex NS2B-NS3. Open arrows indicate cleavage sites, releasing functional viral proteins in the cytosol of the cell. Dark arrows indicate host signalase cleavage sites. Open triangle indicates furin cleavage site and dark triangle indicates unknown host protease cleavage site. TEWETGQI epitope was inserted at two different positions (C): between *f *and *g *strands of the YF 17D virus E protein Domain II located at 482 amino acid of (left panel) or between duplicated flanking viral protease cleavage sites (GARR/S Glycine-Alanine-Arginine-Arginine-Serine) at the junction between NS2B and NS3 proteins (open arrows; right panel) located at 1486 amino acid of the YF 17D virus polyprotein.

To properly express the TEWETGQI epitope between NS2B and NS3 we firstly endowed this sequence with flanking viral protease cleavage site motif. These flanking clevage sites promote the correct epitope release in the citoplasm during viral polyprotein translation and proteolytic processing, yielding the peptide SPTEWETGQIGARR (Figure 1). This recombinant virus, called YF17D/NS2B3/Tc, was recovered after Vero cell transfection indicating that the viral polyprotein was correctly processed, resulting in viable viruses which were able to disseminate in Vero cell culture. The insertion of the TEWETGQI epitope in the *fg *loop of domain II of the E protein did not require any additional sequence, as previously reported elsewhere for different viruses expressing distinct *Plasmodium sp *epitopes in this area of the E protein [14-16]. The recombinant viruses, denominated YF17D/E200/Tc and YF17D/NS2B3/Tc, produced similar post-transfection titers, around 6.0 log_10 _PFU/mL. The presence and integrity of the insert in the viral genome were confirmed by nucleotide sequencing of viral RNA extracted from the stocks.

### Viral proliferation in Vero cells

**Figure 2:**
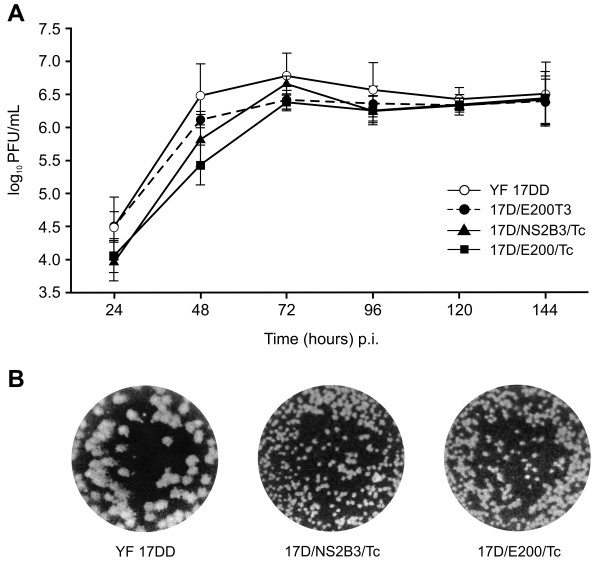
**Characterization of recombinant YF 17D viruses**. A) Replicative kinetics in Vero cells of recombinant viruses. YF 17D/NS2B3/Tc (open triangles) and YF 17D/E200/Tc (black squares), control YF 17D/E200T3 (dark gray discontinuous circles) and YF 17DD (light gray complete circles). Each time point represents the average titer obtained from three separate experiments with the respective standard deviations. No statistically significant differences were found. B) Plaque morphology of YF 17DD, 17D/NS2B3/Tc and YF17D/E200/Tc.

The recombinant viruses, YF 17D/NS2B3/Tc and YF 17D/E200/Tc, were analyzed for their growth properties in Vero cells in comparison to two other control viruses, the YF 17DD vaccine virus and the parental YF 17D/E200T3 virus [14]. Three independent experiments were carried out with a MOI of 0.02 (Figure 2A). The peak titer for all viruses was 72hs post-infection with the maximum virus yields for the vaccine control virus YF 17DD (6.79 ± 0.34 log_10 _PFU/mL). However, the peak titer values obtained from the parental control virus YF 17D/E200T3 (6.42 ± 0.14 log_10 _PFU/mL) or the recombinant viruses YF17D/E200/Tc (6.38 ± 0.12 log_10 _PFU/mL) or YF17D/NS2B3/Tc (6.68 ± 0.12 log_10 _PFU/mL) were very close to the vaccine YF 17DD virus. Hence, the differences among them were not statistically significant (*P *> 0.05, Tukey's test). However, the yield of the recombinant YF17D/E200/Tc up to 48 h post-infection was lower when compared to the other viruses. Figure 2B shows reduced plaque size of both recombinant viruses as compared to parental YF 17DD.

### Epitope expression by the recombinant viruses

**Figure 3:**
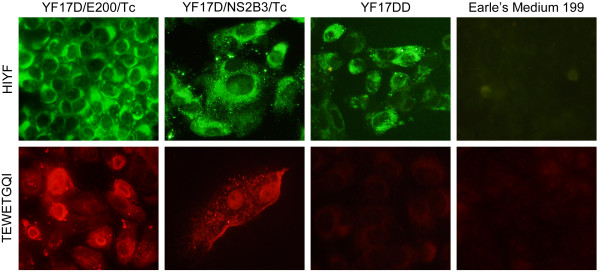
**Expression of the T. cruzi TEWETGQI epitope by recombinant YF17D/E200/Tc and YF17D/NS2B3/Tc viruses**. Indirect immunofluorescence assay of Vero cells infected with recombinant viruses, YF 17DD vaccine virus or not infected (Earle's Medium 199) stained with a mouse polyclonal hyperimmune serum to YF17D (HIYF) or polyclonal antibody directed to the TEWETGQI epitope. Second antibodies were Alexa Fluor 488 goat anti-mouse IgG and Alexa Fluor 546 goat anti-mouse IgG for HIYF (green color) or anti-TEWETGQI (red color) preparations, respectively.

In order to confirm the expression of the eight amino acid *T. cruzi *epitope (TEWETGQI) by the YF 17D/E200/Tc and YF 17D/NS2B3/Tc recombinant viruses, we performed an indirect immunofluorescence assay using a mouse polyclonal serum against TEWETGQI and a mouse polyclonal hyperimmune serum directed to YF 17D (Figure 3). While the YF hyperimmune serum could detect YF antigens in all infected Vero cell conditions, the polyclonal anti-TEWETGQI antibody was only able to stain Vero cells which were infected either by the YF 17D/E200/Tc or YF 17D/NS2B3/Tc viruses, suggesting the correct expression of the TEWETGQI epitope by both recombinant viruses.

### Genetic stability

To evaluate the stability of the foreign insertion expressed by the two recombinant YF viruses, we have performed two series of separate passages in Vero cells per virus sample, infecting cells with a MOI of 0.02. At the second, fifth and eighth passages we performed a nucleotide sequencing analysis of the respective viruses. The TEWETGQI coding sequence could be detected in all samples, either in the intergenic NS2B/NS3 region or the E protein *fg *loop, indicating that both viruses were genetically stable. Furthermore, we plaqued the recombinant viruses belonging to the second, fifth and eighth passages and no variation in plaque size was observed. In addition, full-genome sequencing of viruses in each above passages, failed to reveal any nucleotide differences further supporting the plaquing data.

### Virus attenuation in Swiss mouse model

YF 17D viruses display a considerable degree of neurovirulence for mice [27]. Even though the mouse neurovirulence test does not predict virulence or attenuation of YF viruses for man, it is adopted to assess the fitness of the recombinant viruses as compared to the human vaccine YF 17D virus. This phenotypic trait is verified by intracerebral inoculation of Swiss mice and scoring mortality and average survival time as exhibited in Table 1. The recombinant viruses were significantly less lethal than the vaccine YF 17DD virus, which killed 29 out of 30 animals with an average survival time (AST) of 10 ± 1.8 days, in contrast to 8 out of 30 for the YF17D/NS2B3/Tc and 1 out of 30 for YF17D/E200/Tc, which exhibited AST values of 12 ± 4.1 days and 13 ± 0.0 days, respectively. The differences in mortality rates across groups after 21 days were statistically significant (logrank test; *P *< 0.0001). The animals inoculated with YF17D/E200/Tc experienced a significant lower mortality comparing to those inoculated with the YF17D/NS2B3/Tc (logrank test; *P *< 0.0001). These results indicate that the recombinant viruses exhibit an increased degree of attenuation in comparison to the 17DD vaccine counterpart.

**Table 1:** Swiss mouse neurovirulence of YF 17D viruses

*Inoculation*	*Dose* *(log_10 _PFU)*	*Mouse challenge* *% mortality (dead/tested)*	*AST* *± SD (days)^b^*
Control ^a^	-	0 (0/30)	-
YF 17DD (vaccine)	3.0	97 (29/30)	10 ± 1.8
YF17D/NS2B3/Tc	3.0	27 (8/30)*	12 ± 4.1
YF17D/E200/Tc	3.0	3 (1/30)*	13 ± 0.0

^a ^The mouse control group was inoculated with only Earle's 199 medium by the intracerebral route

^b ^AST, Average Survival Time ± Standard Deviation (SD)

* *P *< 0.05, logrank test (Mantel-Cox) test

### Susceptibility of A/J mice to YF 17DD virus infection

Previous studies have indicated that the A/J (H-2K^k^) mouse is an attractive model for Chagas disease prophylactic studies, since this strain is highly susceptible to infection by *Trypanosoma cruzi*, as are not BALB/c or C57BL/6 lineages [28]. H-2K^k ^mice present parasitemic peaks from 10^th ^to 14^th ^days followed by 100% of death before the 30^th ^day after the challenge [28]. However, we did not know if this specific mouse lineage was susceptible to the YF 17D virus infection. For this purpose, we vaccinated the A/J mice with two doses of the YF 17DD vaccine virus and challenged two weeks later with an intracerebral inoculation of the same virus. The YF 17DD virus immunized mice were completely protected against a lethal YF 17DD virus intracerebral challenge as well as the group of animals which was vaccinated with the recombinant YF17D/NS2B3/Tc virus (Table 2). In contrast, the mock immunized mouse group, that was not protected, died from the 9 to 13 day after YF 17DD challenge.

**Table 2:** Immunogenicity of recombinant YF 17D viruses in A/J mice

*Immunogen ^a^*	*Animals (n)*	*PRNT_50_^a^*		*Protection after YF17D challenge (%)*
		1 dose	2 doses	
YF 17DD	15	358 ± 119	1007 ± 243	100
YF17D/E200/Tc	15	41 ± 12*	124 ± 67**	NT^b^
YF17D/NS2B3/Tc	15	90 ± 20*	274 ± 240**	100
199 Earle's Medium	15	< 10	< 10	-

^a^Reciprocal of the dilution yielding 50% plaque reduction due to neutralizing antibodies in the serum analyzed. Values represent mean of three independent experiments (pooled serum) ± standard deviation (SD). * first and **second dose titers displaying statistically significant differences related to YF17DD group (*P *< 0.05, Tukey's test)

^b ^NT; Not tested

### Immunogenicity of different recombinant YF 17D virus in A/J mice

We have next examined whether recombinant YF 17D viruses bearing the *T.cruzi *epitope would elicit the characteristic neutralizing antibody response. The immunization of the A/J mice with either YF 17DD vaccine virus or any of the recombinant viruses elicited YF neutralizing antibodies (Table 2). After the two-dose regimen, nevertheless, the antibody neutralizing titers for the recombinant viruses (1:124 for YF17D/E200/Tc virus and 1:274 for YF17D/NS2B3/Tc virus) were lower than those obtained with the vaccine YF 17DD virus immunization, which was 1:1,007 (*P *< 0.05, Tukey test). We could not observe any significant difference in the antibody neutralizing titers induced by the recombinant virus immunizations (*P *> 0.05, Tukey test). These data suggest again an increased attenuation of the recombinant viruses.

### Vaccination with recombinant YF 17D viruses induces IFN-γ mediated-cellular immune responses

**Figure 4:**
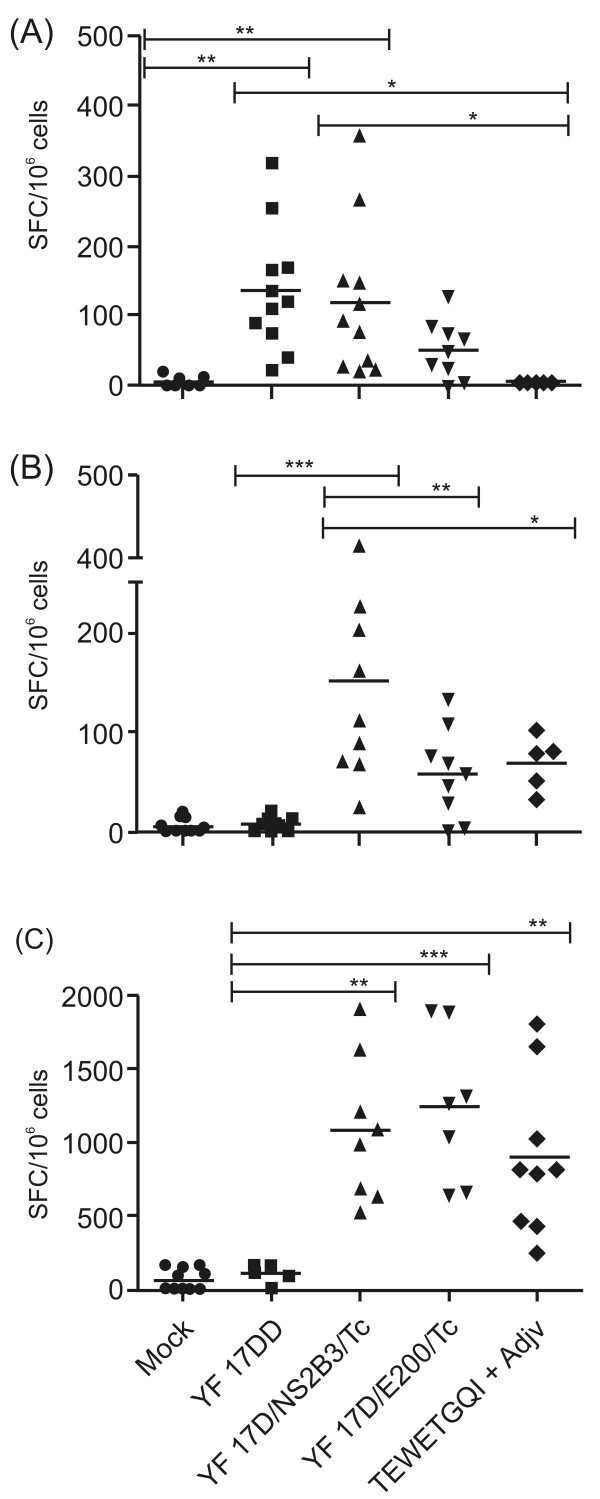
**ELISPOT assay for TEWETGQI-specific IFN-γ secreting cells before and after a *T. cruzi *challenge**. Groups of A/J mice were immunized with Mock (Earle's 199 Medium), YF17DD virus, recombinant viruses (YF17D/E200/Tc and YF17D/NS2B3/Tc) or with TEWETGQI emulsified in Freund's Adjuvant. Spleen cells were obtained one week after the last dose (before challenge) or two weeks after challenge. A) Spleen cells response to YF17DD stimulus before challenge. B) Spleen cells response to TEWETGQI stimulus before challenge. C) Spleen cells response to TEWETGQI stimulus after challenge. Results represent IFN-γ producing cells (SFC) per 10^6 ^spleen cells. Statistically significant differences (ANOVA Tukey's test) are shown in the graphics.

Having previously shown that recombinant YF 17D viruses were capable of eliciting a significant neutralizing response to YF, the next step was the analysis of the immunogenicity of the *T.cruzi *TEWETGQI epitope recombinant viruses in A/J mice. For this purpose we evaluated by ELISPOT the number of specific gamma interferon (IFN-γ**) **producing cells recovered from the spleen of mice that were previously immunized or not with YF 17DD vaccine, the two YF 17D recombinant viruses and with the *T. cruzi *epitope TEWETGQI emulsified in Complete Freund's Adjuvant (Figure 4A and 4B) or immunized and challenged with *T. cruzi *(Figure 4C). First, we stimulated splenocytes *in vitro *with inactivated YF 17DD virus (Figure 4A) to assess cellular immune response against YF structural epitopes mainly. We found that YF 17DD and YF17D/NS2B3/Tc are specifically inducing gamma interferon-cells production as compared to either Mock or TEWETGQI-Adjuvant immunized groups (*P *< 0.05, Tukey's test). Nevertheless, YF 17D/E200/Tc virus induced about 52 ± 41 (mean ± SD) SFC/10^6 ^cells while YF 17D/NS2B3/Tc induced 119 ± 108 SFC/10^6 ^cells, similar to YF 17DD (136 ± 88 SFC/10^6 ^cells). Although differences between YF17D/E200/Tc and YF 17DD immunized groups or between recombinant groups were not statistically significant, YF17D/E200/Tc seem to be a borderline virus since we can not statistically distinguish this immunized group from Mock.

When we evaluated the specific CD8^+ ^T cell response due to the TEWETGQI stimulus (Figure 4B), we detected significant differences in the number of IFN-γ producing cells (SFC) in the group vaccinated with YF17D/NS2B3/Tc recombinant virus in comparison to the vaccinated YF17DD group (*P *< 0.05, Tukey's test). However, there were no statistically significant differences among YF17D/E200/Tc, TEWETGQI-Adjuvant and control YF17DD groups. The YF17D/NS2B3/Tc virus elicited a mean of 151 ± 119 SFC/10^6 ^cells while the YF17D/E200/Tc virus and TEWETGQI control groups induced 57 ± 44 and 68 ± 27 SFC/10^6 ^cells, respectively (differences between recombinant groups and among YF17D/NS2B3/Tc and TEWETGQI groups were significant, *P *< 0.05, Tukey's test).

Next, we evaluated specific CD8^+ ^T cell expansion after a *T. cruzi *trypomastigote challenge (Figure 4C) in vaccinated mice. There proved to be a significant increase in the levels of IFN-γ production for both recombinant viruses, with a mean of 1,077 ± 490 SFC/10^6 ^cells for YF 17D/NS2B3/Tc group and 1,240 ± 514 SFC/10^6 ^cells for YF 17D/E200/Tc. The TEWETGQI-adjuvant immunized group exhibited a mean of 999 ± 527 SFC/10^6 ^cells. Differences of these three groups were statistically significant in contrast to the YF 17DD group (*P *< 0.05, Tukey's test). Despite the lower levels of IFN-γ producing cells induced by the YF17D/E200/Tc in the pre-challenge tests shown above, this virus were able to induce similar levels of IFN-γ producing cells as YF17D/NS2B3/Tc and the TEWETGQI-adjuvant groups in post-challenge tests. There were no differences among recombinant viruses and TEWETGQI-adjuvant condition (*P *> 0.05; Tukey test).

These results clearly demonstrate that these CD8^+ ^T cells were primed during immunization of mice with recombinant YF 17D viruses and expanded after *T. cruzi *challenge. This expansion was 20 times higher for YF17D/E200/Tc. Moreover, in control groups there was no anamnestic response to TEWETGQI after a trypomastigote challenge as observed with the groups immunized with the recombinant viruses (YF17D/E200/Tc or YF17D/NS2B3/Tc).

### Vaccination with 17D recombinant viruses does increase mice survival time after a lethal challenge with *T. cruzi*

**Figure 5:**
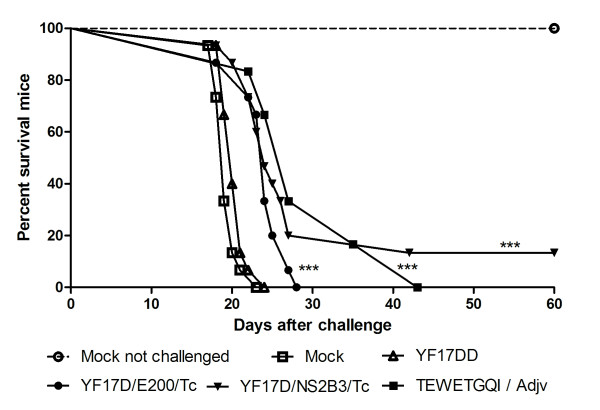
**Kaplan-Meier survival curves**. Groups were immunized twice with medium (Mock), YF 17DD, the two recombinant viruses or with TEWETGQI emulsified in Freund's Adjuvant and infected or not intraperitoneally with 250 *T. cruzi *bloodstream trypomastigotes. Differences between the recombinant viruses and the YF17DD groups were statistically significant (*** *P *< 0.0001, logrank test). Results represents pooled data obtained from three independent experiments with 5 animals per group per experiment.

In order to establish the degree of protection induced by immunization with YF17D recombinant viruses, we used a challenge dose of 250 trypomastigotes of the *T. cruzi *Y strain, which led to a 100% mortality rate after 23 days post-inoculation (dpi) in the A/J mice. However, the immunization with YF17D/E200/Tc or YF17D/NS2B3/Tc viruses reduced mouse mortality after challenge, displaying significant levels of protection as we also detected in TEWETGQI-adjuvant animals, in comparison to YF 17DD and Mock-infected controls (*P *< 0.0001, logrank test) (Figure 5). Notwithstanding, we could not observe complete mouse protection, since only two out of fifteen (13% of protection) survived in the YF17D/NS2B3/Tc immunized group until the day 60 (Table 3). These surviving mice displayed clinical symptoms of parasitic infection (fever, loss of apetite, apathy), which entirely recovered around 25 dpi (data not shown). Moreover, recombinant virus groups presented an average of survival time (AST) longer than the YF17DD group, as YF17D/NS2B3/Tc group presented an AST of 24.8 ± 5.8 (*P *= 0.0005, Mann-Whitney test) and YF17D/E200/Tc group, an AST of 23.7 ± 2.9 (*P *= 0.001, Mann-Whitney test). No significant differences were found between the recombinant viruses average survival times, although YF17D/NS2B3/Tc immunization and the TEWETGQI-adjuvant control group seems to provide a longer survival time than YF17D/E200/Tc (Table 3).

**Table 3:** Protection of vaccinated A/J mice after a *T. cruzi *challenge

*Immunogen*	*Days after challenge* *live/total animals (% survival)^a^*			*AST ± SD*
	23dpi	40dpi	60 dpi	
Mock	0/15 (0)	0/15 (0)	0/15 (0)	19.3 ± 1.4
YF 17DD	1/15 (6)	0/15 (0)	0/15 (0)	20.3 ± 1.5
YF17D/E200/Tc	10/15 (66)	0/15 (0)	0/15 (0)	23.7 ± 2.9**
YF17D/NS2B3/Tc	9/15 (60)	3/15 (20)	2/15 (13)	24.8 ± 5.8***
TEWETGQI/Adjv	5/6 (83)	1/6 (16)	0/6 (0)	29.7 ± 7.9***

^a ^Mice were immunized with medium (Mock), YF 17DD, the two recombinant viruses or with TEWETGQI emulsified in Freund's Adjuvant. Four weeks after the second dose, mice were challenged i.p. with 250 bloodstream trypomastigotes. Average survival times (AST) were significant different (*** *P *< 0.0005, ** *P *< 0.001, Mann Whitney test).

## Discussion

Due to the lack of a current chemotherapy to Chagas disease as other neglected disease, it is essential to develop new strategies for its prevention. For the past decade, several *Trypanosoma cruzi *antigens have been tested for protective immunity of mice. They have included plasmid DNA, recombinant proteins associated with several adjuvants and bacteria or virus vectors [24,25,29,30]. However, DNA vaccine immunogenicity is not yet well established in man, despite the fact that recombinant protein or subunit vaccines may require the use of adjuvant formulations certified for human use [31].

The Yellow fever vaccine virus (YF 17D) has been successfully used as a viral platform to express antigenic sequences from other flaviviruses [11], non-related pathogens such as SIV [32,33], *Plasmodium spp*. [13,14,16,34] and influenza [35,36]. The recombinant YF 17D viruses described here are replication-competent viruses with advantages over other viral vectors such as lifelong immunogenicity and no integration into the host genome. Importantly, in our studies, we demonstrated that pre-existing neutralizing antibodies to YF 17DD (elicited after the initial dose) did not hamper immune responses after a second dose of vaccination, as measured by antigen-specific IFN-γ producing cells and higher YF-specific neutralizing antibody titers.

Considering these favorable characteristics of the YF 17DD vaccine virus and based on the previous protection results reported with the antigens present in the intracellular forms of the parasite [37], we decided to express an epitope of *T. cruzi *Amastigote Surface Protein 2 (ASP-2). The selected TEWETGQI motif is an immunodominant CD8+ T cell epitope, which binds to the MHC class I H-2K^k ^molecule [25]. To express this *T.cruzi *octapeptide, we employed two distinct methodologies. In the first, the insertion in the intergenic NS2B-NS3 region, it is expected to occur that, after the proteolytic cleavage of part of viral precursor polyprotein mediated by the viral NS2B-NS3 proteolytic complex, the foreign peptide will be released in cytoplasm and be presented by MHC class leading to CD8+ T cell activation. Using this approach, it was possible to demonstrate that mouse vaccination with single dose of different recombinant yellow fever viruses could promote partial or complete protection against a challenge with a rodent malaria parasite or a lethal melanoma, respectively. In both studies, the protection was associated to the induction of IFNγ-secreting CD8^+ ^T cells [12,13]. The second approach involved the epitope expression on the surface of viral E protein, the major flaviviral envelope protein. Besides being the main target of viral neutralizing antibodies, several CD8+ T cell epitopes have been mapped in YF 17D E protein in mice, rhesus macaques and humans [7,17,18]. Akondy and colleagues [7] verified that more than ten percent of total IFN-gamma producing CD8+ T cell in vaccines were specific to epitopes present in E protein. It is conceivable that processing of epitopes via MHC class I occurs through either virion endocytosed virus particles or ER-phagosome interaction [38].

Therefore, the expression of the TEWETGQI epitope at two different sites of a vector known to elicit strong CD8+ T cell immune response, with likely different pathways for processing and presentation, was approached in terms of its importance in protection against challenge with live trypanosomes. This is the first description of recombinant YF 17D viruses expressing an antigen of *T. cruzi*, the causative agent of Chagas disease. These recombinant viruses were characterized with regard to the foreign epitope expression, viral growth, attenuation and immunogenicity for mice. Both viruses replicated well in Vero cells, producing similar titers to YF 17DD at 72 hours post-infection and were capable to maintain the heterologous epitope insertions until at least the eighth serial passage in Vero cells. However, insertions clearly affected viral plaque morphology in Vero cells, when compared to the vaccine virus plaque phenotype. Both recombinants were also less virulent for mice after intracerebral inoculation than the YF 17DD vaccine virus. While YF 17DD killed most mice, only about one third of the animals died after the YF17D/NS2B3/Tc inoculation and a yet fewer died with YF 17D/E200/Tc. With regard to the induction of neutralizing antibodies after only one dose, the titers elicited by the recombinant viruses were significantly lower than those elicited by the YF 17DD. After the second dose, inferior titers are still evident for both recombinant viruses. Although we could not find any statistically difference between the recombinant viruses, YF17D/E200/Tc seemed to induce lower titers of neutralizing antibodies than YF17D/NS2B3/Tc. It is believed that this insertion in the *fg *loop of the E protein may affect the conformational change ocurring in the trimerization of this protein at the process of fusion to the endosome membrane which might result in a more attenuated phenotype or increased susceptibility to viral neutralization [16].

An unusual lineage of mice named A/J (H2-K^k^) was adopted in this work to evaluate YF immune responses due to its high susceptibility to *T. cruzi *infection [28]. We observed that immunization with the YF 17DD virus successfully protected mice of this lineage against a YF 17DD intracerebral challenge and also elicited titers of neutralizing antibodies against YF 17DD as described for BALB/c mice [39]. It has already been demonstrated that every YF viral protein is immunogenic and possesses CD8^+ ^T cell epitopes [6,7]. As for A/J mice we also demonstrate that this lineage is capable of responding with specific IFN-γ SFC to YF 17DD, this result being comparable to those induced by the two recombinant viruses.

Immunity elicited by ASP-2 is associated with a type I response generated by IFN-γ producing CD4+ and CD8+ T cells [40,41]. High levels of IFN-γ have been associated with protection for many infections by intracellular parasitic and viral agents [42,43]. Former studies showed that IFN-γ secretion by CD8^+ ^T cells was particularly important for survival of BALB/c and C57BL/6 mice after day 14 post infection with *T. cruzi *[44]. The observed levels of protection elicited by both recombinants might be correlated with expansion of IFN-γ production by CD8^+ ^T cells as demonstrated by ELISPOT on day 15 after challenge. We consider this result important as we know that any delay in CD8^+ ^T cell expansion (and gamma interferon production) represents a major factor in *T. cruzi *infection outcome in mice and Chagas disease in man [40,42,44]. We also found that the period between 14 and 23 dpi is also critical for A/J mice survival as almost all naïve (mock) infected animals died by day 23 dpi as opposed to vaccinated animals. The immune response elicited by either recombinant virus controlled incipient infection, leading to partial protection afterwards in contrast to the naïve group. However, more experiments would be needed to determine whether this cellular response is able to persist for a longer period of time after immunization.

A plausible explanation for an incomplete protection may be related to the fact that immunization with this single peptide may not be sufficient to fully protect mice against T. cruzi [44] despite TEWETGQI-specific IFN-γ producing CD8+ T cells [25]. Furthermore, low levels of protection afforded by vaccination with T. cruzi octapeptide correlated with a lower priming of memory peptide-specific T cells generation [26]. In this regard, a broader repertoire of epitopes present in a larger ASP-2 insert may be required to increase the protection since larger portions of ASP-2, employed either as recombinant protein or expressed by plasmids or adenovirus vectors, in different vaccination protocols shown to induce protection against T. cruzi challenge in the range from 80% to 100% [25,26,30]. In addition, the presence of other CD4+ T cell epitopes may be essential in order to contribute to the enhancement of the CD8+ T cell response, as observed for Chagas disease [44] or malaria [34].

Recombinant YF 17D viruses have been considered as a promising approach for immunization against other diseases, particularly due to its polyfunctional immune response [9]. Since a prophylactic strategy for Chagas disease remains to be established, our results on the expression and immunogenicity of *T. cruzi *CD8+ T cell epitope between the NS2B and NS3 proteins of the recombinant YF 17D virus warrant further development. Future viral formulations containing different YF constructs at the NS2B-3 site delivering distinct epitopes against the same pathogen might enhance protective responses. In addition, a YF construct that encompasses a larger ASP-2 segment inserted between the E protein and the nonstructural NS1 protein should also be tried as this site seems to tolerate larger fragments, while retaining fitness and immunogenicity [33,39].

## Conclusions

We were able to express a CD8^+ ^T cell epitope, TEWETGQI, derived from the *Trypanosoma cruzi *amastigote surface protein 2 (ASP-2) in two distinct regions of the YF genome: in the intergenic region of the NS2B and NS3 viral protease and at the E protein dimer surface. Both epitope insertion methodologies were previously characterized. They allowed the correct processing of the viral polyprotein and did not compromise viral viability, being genetically stable up to the 8^th ^passage. The recombinant viruses were less virulent for mice after a intracerebral inoculation than the YF 17DD, confirming that both recombinant viruses have an increased attenuation.

Both YF recombinant viruses elicited titers of neutralizing antibodies to YF 17DD or presented protection against an intracerebral YF challenge in A/J mice. They were also able to elicit YF 17DD or TEWETGQI-specific IFN-γ-producing CD8^+ ^T cells and to expand TEWETGQI-stimulated cells vigourously after a *T. cruzi *challenge. However, recombinant virus possessing the epitope at the E protein seemed to induce significantly lower numbers of IFN-γ-producing cells than the NS2B/NS3 recombinant virus. Although both recombinants were able to delay mortality in *T. cruzi *infected mice and to increase significantly survival, immunization with NS2B/NS3 virus allowed a longer average survival time than the epitope containing E protein virus. Moreover, NS2B/NS3 virus provided a partial mice protection up to the 60^th ^day after challenge.

This system is likely to be useful for a broader live attenuated YF 17D virus-based vaccine development for other human diseases. Besides, insertion of foreign genes into distinct regions of flavivirus genome may also allow further studies on immunologically protective responses associated with specific cell-compartment harboring heterologous epitopes derived from other viral or parasitic agent. It remains to be seen whether expression of larger domains of ASP-1, which include the TWETGQI epitope, will elicit better T-CD8+ responses to the latter. It is likely that additional antigens and recombinant virus formulations will be necessary to generate a protective response.

## Methods

### Mice

Female 4 to 6 week-old A/J mice in this study were obtained from CECAL, Fiocruz (Rio de Janeiro, Brazil) or purchased from CEMIB/UNICAMP. Three week-old female Swiss mice were purchased from CEMIB/UNICAMP. All animal experimentation was in accordance with the Institutional Committee for Experimentation and Care of Research Animals (CEUA-Fiocruz: L-0032) guidelines.

### Cell culture and vaccine virus

Vero cells (ATCC) were grown in Earle's199 medium supplemented with 5% fetal bovine serum (FBS). Vaccine sub-strain YF17DD, used in this study, was reconstituted in 0.5 mL of sterile water as specified by the manufacturer's (Bio-Manguinhos, Rio de Janeiro, Brazil). Vaccine YF17D substrains variability and history of passages was previously described [45], [46]. Vaccine strain YF17 D, used in this study, was reconstituted in 0.5 mL of sterile water as specified by the manufacturer's (Bio-Manguinhos, Rio de Janeiro, Brazil).

### Construction and recovery of parental and recombinant viruses

The parental YF17D/E200T3 virus was constructed and recovered as described elsewhere [14]. This virus contains an *Eco *RV insertion site at nucleotide 1568 of the viral genome. The strategy of insertion between the NS2B/NS3 region was based on the approach previously described [12] with some minor modifications. Briefly, the plasmid pT3 containing the central portion of YF genomic complementary DNA was mutagenized to create a *Sma *I restriction site at the intergenic NS2B/NS3 region using the QuikChange II Site-Directed Mutagenesis Kit (Agilent Technologies) in the presence of mutagenizing oligonucleotides containing the insertion. This mutation inserted a CCC codon at the 4574 YF genome position. The recovery of parental YF17D/NS2B3 virus was made through the two-plasmid system with the pYFE200 plasmid as a source of the ends of genomic YF DNA as previously described [14]. To obtain the recombinant viruses, the plasmids pYFE200 and *Sma *I mutagenized pT3, were digested with *Eco *RV and *Sma *I, respectively to insert the TEWETGQI epitope (ASP-2 protein amino acids 320 - 327). Synthetic dephosphorylated oligonucleotide 5' ACAGAATGGGAGACAGGACAGATC 3' and its complement were annealed and ligated, into the *Eco*RV restriction site of pYFE200 (to generate YF17D/E200/Tc virus) or into the *Sma *I site of pYFT3 (to generate YF17D/NS2B3/Tc virus). After chemically competent *Escherichia coli *MC1061 transformation, recombinant plasmids were screened by digestion with *Eco*RV (pYFE200) or *Sma I *(pT3) followed by nucleotide sequencing to confirm insertion orientation. Two sets of templates were generated by *Sal *I and *Nsi *I cleavages and T4 DNA ligase procedure. The first consisted on the ligation of the recombinant TEWETGQI pYFE200 plasmid with the pT3 plasmid fragments. The second was the ligation of the pE200 plasmid plus the recombinant TEWETGQI pT3 plasmid. All templates were linearized by *Xho *I digestion and submitted to *in vitro *transcription by SP6 RNA polymerase (AmpliScribe SP6 kit, Epicentre Technologies). The viral RNA was transfected into Vero cells with LipofectAmine (Invitrogen), as previously reported [14]. After the onset of cytopathic effect, the viral supernatant was collected and prepared for a second passage viral stock for all subsequent experiments.

### Viral growth in Vero cells

Viral proliferation curves were determined by infecting monolayers of Vero cells at MOI of 0.02. Cells were seeded at a density of 62,500 cell/cm^2 ^and infected 24 hs later. Samples of the cell culture supernatant were collected at 24-hour intervals post-infection. Viral yields were estimated by plaque titration on Vero cells. The different viral growth peaks were compared using the *One Way ANOVA *test. The Tukey test was selected as a posttest to compare pairs of group means. (GraphPad Prism 5.03 Program). The differences were only considered significant when *P *< 0.05.

### RT/PCR and sequencing

Viral RNA was extracted with Trizol LS (Invitrogen) and used as a template for cDNA synthesis as previously described [14]. Two sets of YF-specific synthetic oligonucleotides were employed. To amplify the sequence containing the NS2B/NS3 region, a sense primer encompassing the genomic position 4,181 to 4,200 nt and a reverse from 4,858 to 4,874 were utilized. The RT-PCR fragment containing the envelope insertion site was obtained using the positive sense primer and the reverse primers corresponding to nucleotides 940 to 960 and 1,781 to 1,799. After QIAquick PCR Purification (QIAGEN), PCR products were submitted to nucleotide sequencing as described elsewhere [39]. Nucleotide sequences were analyzed with Chromas software version 2.3 (Technelysium Pty Ltd) and Seq-Man II software from Lasergene package version 8.0 (DNAStar Inc.).

### Genetic stability assay

Recombinant viruses were submitted to two independent series of eight passages in Vero cells at MOI of 0.02. In the second, fifth and1) eighth passage, viral RNA was extracted at 72 hs post-infection from the Vero cell culture and submitted to RT/PCR and sequencing as above.

### Mouse neurovirulence studies

Groups of ten three-week old Swiss mice were inoculated by the intra-cerebral route as previously described [14]. Animals were followed up for 21 days and deaths recorded. Cumulative mortality and survival curves (Kaplan-Meyer method) were compared across groups and formally tested using the logrank test (GraphPad Prism 5.03 Program).

### Fluorescence microscopy

Vero cells monolayers were infected at a MOI of 0.1 with Earle's199 medium alone, control virus YF 17DD or recombinant viruses as previously described [14]. We adopted as primary antibodies mouse anti-TEWETGQI or mouse hyperimmune ascitic fluid to YF 17D (ATCC). Mouse anti-TEWETGQI antibodies were generated by immunization of BALB/c mice with a recombinant fusion protein containing the TEWETGQI fused to the glutathione S-Transferase. Mice were immunized with the 25 μg of the recombinant protein emulsified in Complete Freund's Adjuvant and subsequently boosted twice with the same amount of protein emulsified in Incomplete Freund's Adjvant [47]. This anti-sera was used a final dilution of 1:200. As secondary antibodies, we used Alexa Fluor 546 goat anti-mouse IgG-Invitrogen or Alexa Fluor 488 goat anti-mouse IgG-Invitrogen (diluted 1:400), according to the manufacture's protocol. Both preparations were treated with SlowFade-Gold antifade reagent with DAPI (Invitrogen) and analysed by Fluorescence Microscopy with an Olympus IX51 Inverted Microscope.

### Immunogenicity of YF 17D virus in A/J mice

Groups of five 4-week old A/J mice (H-2K^k^) were subcutaneously injected with Medium (Mock) or with 100,000 PFU of YF 17DD virus or YF 17D recombinant viruses on days 0 and 15. An additional group received two doses of the TEWETGQI epitope diluted in PBS and emulsified in Freund's adjuvant (Sigma) according to specifications of the manufacturer in a final concentration dose of 40 μg/50 μL. Two weeks after the second dose, mice were bled (retrorbital) and challenged intracerebrally with 3,000 PFU of YF 17 D. Challenged animals were monitored for 21days and deaths recorded. Serum samples were analyzed for antibodies YF by the plaque-reduction neutralization test (PRNT) as described [14]. Plaque neutralization titers were calculated as the highest dilution of antibody reducing 50% of the input virus plaques. One-way ANOVA was used to compare the mean titers of neutralizing antibodies of the experimental groups. The Tukey test was selected as a posttest to compare pairs of group means. GraphPad Prism 5.03 Program was utilized to analyze the data.

### Protection of vaccinated A/J mice against a lethal *T. cruzi *challenge

To perform *T. cruzi *challenge assays, groups of five 4-6 week-old A/J mice were immunized with the different viruses as described above. Four weeks after the last dose, mice were challenged intraperitoneally with 250 bloodstream trypomastigotes of the *T. cruzi *Y strain. After challenge, clinical symptoms and deaths were daily recorded for a period of 60 days. Results represent pooled data from three independent experiments involving five animals per group and per experiment. Cumulative mortality and survival curves (Kaplan-Meyer method) were compared across groups and formally tested using the logrank test (GraphPad Prism 5.03 Program).

### IFN-γ ELISPOT assays

For the IFN-γ ELISPOT assays, groups of mice were immunized as described above, including a control group of TEWETGQI peptide. One week after the second dose and two weeks after *T. cruzi *challenge, mice were sacrificed and the spleens removed. All assays were carried out using BD IFN-γ ELISPOT set (BD - Biosciences Pharmingen, San Diego-CA). Plates were coated with 10 μg/mL of purified anti-IFN-γ and after overnight incubation, plates were blocked with RPMI 1% FBS for at least 2 hours at 37°C in atmosphere containing 5% CO_2_. Spleen cells were removed aseptically and red blood cells were lysed with hemolysis solution (NH_4_Cl 0.14 mol/L/Tris 0.017 mol/L pH 7.2). Leucocytes were resuspended to a concentration of 2 × 10^5 ^viable cells per mL in RPMI 1640 medium (Invitrogen) supplemented with 1 M HEPES buffer, 2 mM L-Glutamine, 5 μM β-mercaptoethanol, 1 mM sodium pyruvate, 1% non-essential amino acid solution, 1% (V/V) vitamin (all from Invitrogen), 10% (V/V) Fetal Bovine Serum (Gibco) and 50 μg/mL gentamicin. Cells were incubated with Medium alone, 10 μg/mL of synthetic TEWETGQI peptide (Invitrogen), 10,000 PFU/mL of inactivated YF 17DD virus or 4 μg of concanavalin A (Sigma) diluted in RPMI 10% FBS medium. After incubation of 48 hs at 37°C in an atmosphere containing 5% CO_2_, plates were extensively washed and incubated with biotinylated anti-IFN-γ antibody (BD) followed by peroxidase-labeled streptavidin diluted 1:800 in PBS containing 10% FBS. After 3 hours of incubation, plates were washed with PBS-Tween and *spots *were developed by adding AEC BD substrate with cromogen (20 μl/mL) and 30% hydrogen peroxide solution from Sigma (1 μl/mL). Reaction was stopped under running water and IFN-γ secreting cells that appeared as red spots were counted with an Immunospot reader (Cellular Technology Ltd., Cleveland, OH) using the Immunospot Software version 3 by the ELISPOT Platform of PDTIS/Fiocruz - Rio de Janeiro (Lima-Junior *et al*., 2008). Statistical significance of the differences was verified with One-way analysis of variance and Tukey's test.

## Competing interests

The authors declare that they have no competing interests.

## Authors' contributions

RTN carried out the YF virus genome cloning, viral characterization and immunological work, and drafted the manuscript; ARN performed the *T. cruzi *Y strain challenge assays; MCSP participated in the design of the study and was engaged in the animal studies; MMR assisted in data interpretation; RG helped to coordinate the study and manuscript draft; MCB designed the viral constructions, coordinated the study and manuscript draft. All authors read and approved the final manuscript.
